# Characterizing Lymph Node Burden With Elective Unilateral Neck Irradiation in Human Papillomavirus-Positive Tonsil Squamous Cell Carcinoma: Defining the Upper Limits

**DOI:** 10.7759/cureus.27521

**Published:** 2022-07-31

**Authors:** Jared H Hara, Stanley I Gutiontov, Sophia Uddin, Ari J Rosenberg, Alexander T Pearson, Zhen Gooi, Elizabeth A Blair, Nishant Agrawal, Everett E Vokes, Daniel T Ginat, Daniel J Haraf, Aditya Juloori

**Affiliations:** 1 Department of Radiation and Cellular Oncology, The University of Chicago Medicine, Chicago, USA; 2 Department of Radiation Oncology, University of Illinois Hospital & Health Sciences System, Chicago, USA; 3 Department of Radiation Oncology, Radiation Oncology Associates, Mequon, USA; 4 Department of Otorhinolaryngology – Head and Neck Surgery, University of Maryland, Baltimore, USA; 5 Department of Medicine, Section of Hematology/Oncology, The University of Chicago Medicine, Chicago, USA; 6 Department of Surgery, Section of Otolaryngology – Head and Neck Surgery, The University of Chicago Medicine, Chicago, USA; 7 Department of Radiology, The University of Chicago Medicine, Chicago, USA

**Keywords:** intensity-modulated radiotherapy, hpv-related oropharyngeal cancer, quality of life, ipsilateral neck radiation, human papilloma virus, oral and oropharyngeal cancer, head and neck squamous cell cancer

## Abstract

Objectives

Elective unilateral neck irradiation in well-lateralized tonsil carcinoma for N2b disease is controversial. Metrics regarding nodal burden beyond the N-stage to define the upper limit of this de-escalation approach remain limited. We investigated the role of nodal number, level, and volume on outcomes in patients with well-lateralized tonsil carcinoma treated with this approach.

Methods

A total of 37 patients received radiotherapy (RT) with unilateral neck coverage for well-lateralized tonsil cancer. Of patients, 95% had p16+ disease, and 81% were staged with positron emission tomography/computed tomography. The majority of patients received definitive chemoradiation on prospective de-escalation trials. Ten patients had ipsilateral neck dissections and were treated adjuvantly. The median RT dose to the ipsilateral neck (generally II-IV) was 45 Gy. The effects of nodal number, max dimension, volume, and level on recurrence-free survival (RFS) and overall survival (OS) were to be analyzed via Cox proportional hazards (Cox-PH).

Results

After a median follow-up of 3.9 years, two-year RFS and two-year OS were 100% and 97%, respectively. Given the 0% contralateral recurrence rate, Cox-PH analysis was not performed. Of patients, 70% were American Joint Committee on Cancer (AJCC) 7th edition N2b, with a median number of nodes, number of nodal levels, max dimension, and volume of two, one, 3.4 cm, and 15.6 cc, respectively. There were several patients with low-lying nodes; aggregate nodal volume measured was up to 85.4 cc.

Conclusion

Unilateral neck irradiation in well-lateralized tonsil carcinoma resulted in no contralateral recurrence. Nodal volume, level, and number do not seem to have a significant impact on outcomes.

## Introduction

Oropharyngeal squamous cell carcinoma (OPSCC) has an incidence of nearly 100,000 patients and results in nearly 50,000 deaths worldwide annually. Even though tobacco use, a major risk factor for OPSCC, has been decreasing worldwide, there has been an increase in OPSCC cases over the past few decades due to rising rates of human papillomavirus (HPV) infection [1]. Therefore, optimizing the treatment of OPSCC is an important area of investigation.

Typically, the treatment of early-stage tonsil squamous cell carcinoma involves either primary surgery with adjuvant radiation ± chemotherapy or definitive chemoradiation. Radiation therapy planning involves weighing multiple factors to minimize both the risk of recurrence and the side effects of radiation therapy, such as xerostomia and dysphagia [2,3]. Historically, patients with tonsil cancer were treated with elective nodal irradiation to the bilateral necks. With the improvement in technical capabilities provided by intensity-modulated radiation therapy (IMRT), clinicians began exploring reduced treatment volumes in well-selected patients. Prior prospectively collected data have demonstrated improved quality of life [4] and reduced feeding tube rates [5], with a reduction in elective nodal irradiation volumes [6].

One widely adopted approach by O’Sullivan et al. [2,7] showed low rates of contralateral neck failure (3.5%) in patients with well-lateralized tumors treated with unilateral neck radiation. The American Radium Society consensus statements [8] recommend unilateral neck irradiation mainly based on primary tumor extent - well-lateralized tonsil tumors with less than 1 cm of soft palate or base of tongue extension. However, national guidelines vary on the importance of nodal burden on the omission of the contralateral neck from the radiation field [8]. Importantly, O’Sullivan et al. and Huang et al. included a limited number of patients with multiple ipsilateral nodes (five and seven N2b patients, respectively) [2,7]. Thus, there are limited data available to guide clinicians about the risks of contralateral neck failure in patients with the American Joint Committee on Cancer (AJCC) 7th edition N2b disease. Moreover, these studies have not addressed specific metrics with regard to nodal burden beyond the N stage and nodal level. The upper threshold for patient selection for this de-escalation approach, therefore, remains undefined [9].

In the present study, we review institutional outcomes of patients with well-lateralized tonsil cancer treated with unilateral neck radiation between 2010 and 2017. The specific objective of this study was to investigate the potential impact of nodal number, level, and volume on outcomes in our cohort of patients with well-lateralized tonsil carcinoma treated with this approach, hypothesizing that these factors would not impact the risk of contralateral neck failure.

## Materials and methods

Study design and patient population

We performed a retrospective institutional database search for patients with squamous cell carcinoma of the tonsil diagnosed between January 2010 and December 2017 with institutional review board approval and waiver of consent. The study population consisted of diverse patients treated over a long period, most of whom were treated per contemporary de-escalation trial parameters [5,10,11]. Only patients treated to the ipsilateral hemineck with radiation were included in our study. Patients who received prior head and neck radiotherapy for prior head and neck malignancy were not included in this study. Patient demographic, pathologic, and clinical risk factors are summarized in Table 1. Patient age was defined as the age at initial diagnosis. HPV status was identified by immunohistochemical staining or by HPV polymerase chain reaction. The histopathologic review was performed by pathologists and staging was defined using clinical and radiographic features according to the AJCC 7th and 8th editions. Recurrence was defined as any local, regional, or distant metastatic failure using both clinical and radiographic evaluation. Survival was defined as the length of time from diagnosis to the date of last follow-up or the date of radiographic progression or the date of death for recurrence-free survival (RFS) or overall survival (OS), respectively.

**Table 1:** Patient characteristics IQR: interquartile range; KPS: Karnofsky Performance Score.

	N = 37
Age (years), median (IQR)	57.6 (53.5-61.6)
KPS, median (IQR)	100 (80-100)
Sex, No. (%)	
Male	32 (86%)
Female	5 (14%)
Smoking, No. (%)	
Never smokers	15 (41%)
Currently smoking	
Yes	3 (8%)
No	34 (92%)
Pack year history	
Low risk (including non-smokers)	21 (57%)
High risk (>10 pack years)	15 (41%)
Unknown	1 (2%)

Management, radiation treatment, and follow-up

The standard diagnostic pathway comprises a comprehensive workup before starting treatment including history and clinical examination, serum tests, fiberoptic nasendoscopy, and biopsy. Imaging consists of a diagnostic neck CT ± neck MRI as well as fluorodeoxyglucose (FDG) positron emission tomography (PET) combined with CT (PET/CT). Prior to initiating treatment, patients are evaluated in consultation with the surgical team (ENT), medical oncology, radiation oncology, a nurse specialist, a speech and language pathologist, and a dietician, and undergo a dental review. Following an initial evaluation, their cases were reviewed at our weekly institutional multidisciplinary tumor board. Pathology was reviewed by our institutional head and neck pathologists.

The decision to treat with ipsilateral or bilateral radiation followed institutional management policies and was discussed in weekly peer-reviewed quality assurance rounds. Ipsilateral radiotherapy was recommended for N0-N2b disease in lateralized tonsillar primaries limited to the lateral one-third of the “hemi-structure” of the base of tongue or soft palate, defined as ≤ 1 cm of superficial mucosa of “hemi-structure” extension, without muscle involvement or suspicion of deeper penetration. Radiotherapy alone was given for N0-N1 disease, but concurrent chemoradiation (CRT) was preferred for N2a-b disease.

Patients underwent individualized CT-based planning before the beginning of treatment with immobilization in alpha cradles. Patients underwent re-simulation following completion of induction chemotherapy and again at week three of radiation for adaptive re-planning. The clinical target volume (CTV) included treatment to the primary site as well as the neck as determined by the treating radiation oncologist. IMRT was used to treat the volume. Patients were treated with a combination of mono- to tri-modal therapy of upfront surgery, and radiation with or without concomitant chemotherapy. Upfront surgical procedures included either primary surgery or neck dissection. Several patients were treated per de-escalation protocols [5,10,11], which utilized a chemotherapy-response adapted de-escalated treatment. In an earlier volume-based de-escalation protocol [10], patients received induction cisplatin (75-100 mg/m^2^), paclitaxel (175 mg/m^2^), and everolimus (5-10 mg) ± cetuximab (250 mg/m^2^) for two cycles. Patients then received CRT; patients with a response ≥ 50% received treatment to the primary tumor and involved nodes with a 1.5 cm expansion of the gross tumor volume to 75 Gy, while non-responders received treatment to the primary, involved nodes, and the first uninvolved nodal echelon to an elective dose of 45 Gy followed by a boost to 75 Gy. The subsequent dose- and volume-based de-escalation protocol utilized carboplatin (area under the curve = 6) and nab-paclitaxel (100 mg/m^2^) for three cycles [5,11]. Patients then received induction response-based radiation alone or CRT. CRT was utilized for patients with either high-risk disease (>10 smoking pack years, T4, or N2c-N3) or a <30% response by Response Evaluation Criteria in Solid Tumors (RECIST). Patients with no high-risk disease and ≥30% response were eligible for dose-reduced CRT to 45 Gy to the primary site, while low-risk patients with ≥50% were eligible for 50 Gy of radiation alone. Radiation dose/fractionations were treated in either a “conventionally fractionated” (daily, 1.8-2.0 Gy fractions) or in week-on-week-off accelerated fractionation (1.5 Gy twice-daily irradiation followed by a nine-day treatment break). Chemoradiation typically consisted of three to five alternating weekly cycles of paclitaxel (100 mg/m^2^ on d1), infusional 5-fluorouracil (600 mg/m^2^/d on d1-5), and hydroxyurea (500 mg oral twice per day) (TFHX). Alternatively, concurrent weekly cisplatin (40 mg/m2) was also utilized. Doses were based upon patient weight and surface area.

Follow-up was undertaken in a multidisciplinary setting. CT or MRI was performed eight to 12 weeks after radiotherapy to assess treatment response. In the later time period of the cohort, patients underwent a planned neck dissection four to eight weeks following dose-reduced radiation treatment as a part of an experimental de-escalation protocol [5]. Routine surveillance was undertaken at three-month intervals for the first two years; four-month intervals in years two to three, six-month intervals for years three to five, and annually thereafter. Local or regional failures were recorded based on histologic confirmation, and distant failure relied on radiologic evidence, histologic evidence, or both.

For the purpose of this study, tumor measurements and volumes were obtained post hoc. Tumor measurements were obtained through an independent review of pre-therapy imaging (CT, MRI, and PET/CT) by a radiation oncologist and neuro-radiologist. Lymph nodes with a short axis ≥ 8 mm were eligible for inclusion in tumor burden estimation. Total nodal volume was obtained via nodal delineation by a radiation oncologist and calculated using the Pinnacle treatment planning system version 9.8 (Philips, Amsterdam, Netherlands).

Statistical analysis

Statistical analyses were performed using STATA version 17.0 (StataCorp LLC, College Station, TX). Descriptive statistics were utilized to characterize clinicopathologic and treatment characteristics. Survival analysis was performed using the Kaplan-Meier method and Cox proportional hazards. The Kaplan-Meier method was used to estimate rates of RFS and OS. Due to the absence of failures, including both locoregional and distant, no statistical analysis was performed to compare rates of RFS and OS.

## Results

Patient characteristics

We identified 37 patients who underwent ipsilateral neck irradiation for ipsilateral tonsillar cancer, and their clinical features are summarized in Table 1. The initial date of diagnosis ranged from January 2010 to February 2017. The median age of diagnosis was 57.6 years, and the median Karnofsky Performance Score (KPS) was 100. Of the patients, 32 (86%) were male, and five (14%) were female. Three (8%) patients were currently smoking at treatment, while 34 (92%) quit. Fifteen (41%) patients were non-smokers. Fifteen (41%) patients had a >10 pack year history, while 21 (57%) had a <10 pack year history (including non-smokers). Thirty (81%) patients underwent upfront PET/CT.

Clinicopathologic features

A total of 35 (95%) patients were HPV+ (Table 2). The maximum nodal tumor diameter was 3.6 cm with a nodal volume of 15.6 cc (interquartile range (IQR): 10.9-21.8). Of the patients, 21 (57%) had ≥ two nodes. Sixteen (43%) patients had two nodes, nine (24%) patients had three nodes, and one (3%) patient had five nodes. The median number of involved nodal levels was one (IQR: 1-3), and 14 (38%) patients had multiple levels involved: nine (24%) patients with two levels involved, three (8%) with three levels involved, and one (3%) with four levels involved. A total of 26 (70%) patients were found to have AJCC 7th edition stage N2b disease.

**Table 2:** Tumor characteristics HPV: human papillomavirus; IQR: interquartile range; AJCC: American Joint Committee on Cancer.

	N = 37
HPV status, No. (%)	
p16+	35 (95%)
P16-	2 (5%)
Primary side, No. (%)	
Left	13 (35%)
Right	24 (65%)
Nodal quantification	
Nodal size (maximum tumor diameter) (cm), median (IQR)	3.6 (2.9-4.1)
Nodal size > 3 cm (maximum tumor diameter), No. (%)	24 (65%)
Nodal volume (cc), median (IQR)	15.6 (10.9-21.8)
Nodal volume in definitively treated patients, median (IQR)	16 (10.9-21.8)
Nodal volume for induction patients, median (IQR)	
Nodal volume (pre-induction)	15.8 (10.9-21.8)
Nodal volume (post-induction)	7.1 (5.4-9.7)
Number of nodes involved, median (IQR)	2 (1-3)
0	1 (3%)
1	10 (27%)
2	16 (43%)
>2	10 (27%)
Number of levels involved, median (IQR)	1 (1-2)
0	1 (3%)
1	23 (62%)
2	9 (24%)
3	3 (8%)
4	1 (3%)
Multiple levels	14 (38%)
Nodal levels involved, No. (%)	
IB	2 (5%)
II	36 (97%)
III	10 (27%)
IV	3 (8%)
V	2 (5%)
Level III, IV, or V involvement, No. (%)	11 (30%)
Level IV or V involvement, No. (%)	3 (8%)
T stage (AJCC 7^th^/8^th^ edition), No. (%)	
1	19 (51%)
2	13 (35%)
3	5 (14%)
N stage (AJCC 7^th^ edition), No. (%)	
0	1 (3%)
1	4 (11%)
2a	6 (16%)
2b	26 (70%)
N stage (AJCC 8^th^ edition), No. (%)	
0	1 (3%)
1	34 (92%)
2b	2 (5%)
Overall stage (7^th^ edition), No. (%)	
III	5 (14%)
IVA	32 (86%)
Overall stage (8^th^ edition), No. (%)	
I	30 (81%)
II	5 (14%)
IVA	2 (5%)

Treatment characteristics

Fifteen (41%) patients underwent upfront primary surgery: 10 (67%) patients had a close/positive margin, and two (17%) patients had a positive margin (Table 3). Ten (27%) patients underwent an upfront neck dissection with a median number of positive nodes of one (IQR: 1-2.25) with a median number of dissected nodes of 24 (IQR: 16-28). Of those patients that underwent neck dissection, three (30%) patients had pathologic extranodal extension (pENE). Two (5%) patients were treated with definitive radiation alone, and 35 (95%) patients received systemic therapy. Ten (29%) patients underwent induction chemotherapy (IC), 11 (30%) patients underwent CRT, and 14 (38%) patients underwent treatment with both IC and CRT. A total of 27 (73%) patients received a median definitive radiation dose of 70 Gy (IQR: 50-74) to the primary and involved nodal region and a median dose to the elective ipsilateral neck of 45 Gy (45-51). Ten (27%) patients received a median adjuvant radiation dose of 66 Gy (IQR: 61-66) to the primary and a median dose to the ipsilateral postoperative neck of 50 Gy (IQR: 50-51.5). Five (14%) patients experienced grade 3+ acute skin toxicity, and 14 (47%) patients experienced grade 3+ acute mucositis, with two (5%) patients requiring a g-tube placement during treatment. Twenty (54%) patients, two (5%) patients, and zero patients experienced late grades 1, 2, and 3 toxicity, respectively.

**Table 3:** Treatment characteristics IQR: interquartile range; TFHX: paclitaxel (100 mg/m^2^ on d1), infusion 5-fluorouracil (600 mg/m^2^/d on d1-5), and hydroxyurea (500 mg oral twice per day); FHX+C: infusion 5-fluorouracil (600 mg/m^2^/d on d1-5), hydroxyurea (500 mg oral twice per day), and cetuximab; FHX: infusion 5-fluorouracil (600 mg/m^2^/d on d1-5), and hydroxyurea (500 mg oral twice per day).

Surgery	N = 15
Primary site, No. (%)	15 (100%)
Pathologic tumor size, median (IQR)	1.4 (1.15-2.58)
Close/positive margin	10 (67%)
Positive margin	2 (17%)
Neck dissection, No. (%)	10 (67%)
Number of positive nodes, median (IQR)	1 (1-2.25)
Number of nodes dissected, median (IQR)	24 (16-28)
% Nodal involvement, median (IQR)	6.00% (3.75-11.75%)
Pathologic extranodal extension, No. (%)	3 (30%)

Chemotherapy, No. (%)	N = 37
Induction	10 (29%)
Concurrent	11 (30%)
Both	14 (38%)
None	2 (5%)
Chemotherapy regimen	
Induction	
TP	9 (28%)
TP+C	3 (9%)
TP+C+/-E	6 (18%)
None	9 (27%)
Concurrent	
TFHX	20 (54%)
FHX+C	4 (10.8%)
FHX	1 (2.7%)
Cisplatin	2 (5.4%)

Radiation	N = 37
Definitive, No. (%)	27 (73%)
Primary and involved neck dose (Gy), median (IQR)	70 (50-74)
Elective dose neck (Gy), median (IQR)	45 (45-51)
Low dose neck (Gy), median (IQR)	45 (36-50)
Adjuvant, No. (%)	10 (27%)
Primary dose (Gy), median (IQR)	66 (61-66)
Elective dose neck (Gy), median (IQR)	50 (50-51.5)
Elective dose neck (Gy), median (IQR)	50 (39-50)

Toxicity, No. (%)	
Acute toxicity	
Skin grade 3+	5 (14%)
Mucositis grade 3+	14 (47%)
G-tube	2 (5%)
Late toxicity	
Grade 1	20 (54%)
Grade 2	2 (5%)
Grade 3	0 (0%)

Follow-up (years), median (range)	3.92 (0.01-9.23)
2-year recurrence-free survival, (%)	100%
2-year metastasis-free survival, (%)	100%
2-year overall survival, (%)	97%
Patients with ≥ 2 years of follow-up, No. (%)	35 (95%)
Planned salvage neck dissection, No. (%)	12 (32%)
Node-positive, No. (%)	0 (0%)

Treatment outcomes

The median follow-up period was 3.92 years (range: 0.01-9.23) (Table 3). A total of 35 (95%) patients were evaluable at two years. Two-year RFS was 100%, with a two-year overall survival of 97%. Zero patients had contralateral neck failure. Twelve patients underwent a planned neck dissection per protocol with no evidence of disease in any of the patients.

## Discussion

Our results demonstrate that ipsilateral nodal disease burden does not predict contralateral neck failure in well-lateralized tonsil cancer patients undergoing radiation therapy. Furthermore, omission of contralateral neck radiation in patients with multiple ipsilateral nodes (AJCC 7th N2b) does not compromise progression-free or overall survival. This is the first report demonstrating that nodal volume, level, and number do not seem to have a significant impact on these outcomes and could, if applied to and corroborated in other series, help increase patient selection for this approach.

Importantly, this study included 26 (70%) patients with N2b disease, a cohort that was under-represented in the initial O’Sullivan et al.'s experience, where only five of 176 patients treated ipsilaterally had N2b disease. Given the lack of supporting data, the most recent American Radium Society (ARS) expert consensus-guided recommendation did not reach a consensus regarding the role of ipsilateral treatment for patients with multi-nodal disease [8]. In fact, in the recently published NRG HN-002 and ongoing NRG HN-005, only patients with multiple nodes limited to level II were allowed to have unilateral neck radiation, and even in this setting, it was only optional [12]. This study includes patients that would not have been candidates for unilateral radiation in ongoing NRG protocols. Of the patients in our cohort, 38% had a multi-station disease, and 10 (27%) patients had three or more nodes involved at diagnosis.

The low risk of contralateral recurrence in our higher-risk cohort is consistent with the majority of retrospective studies available in the literature, as detailed in Table 4 [2,7,13-30]. The largest study by Al-Mamgani et al. consisted of 185 patients treated with IMRT and found a contralateral recurrence rate as low as 1.1% [13]. In contrast, a recent study found a contralateral neck recurrence rate as high as 14.3% (four of 28 patients) in patients with N2b disease treated with ipsilateral radiation [3]. The authors urged caution with this treatment strategy for N2b. However, it is important to note that two of the four patients were successfully salvaged. Overall, when combining patients from our series with patients with N2b status available in the literature, the rate of contralateral neck failure is 1.95% (31 failures of 1590 patients) in the entire unilateral radiation cohort and 4.03% (17 of 422 patients) in the subset of patients with N2b disease (Table 5). Thus, the rates of contralateral recurrence are low, both in the literature and in our series, even in patients with N2b disease.

**Table 4:** Review of the literature on ipsilateral radiation for oropharyngeal cancer ^i ^No low lying nodes; ^ii ^adjuvant study; ^iii ^> or ≤ 3 cm. N: sample size; CNR: contralateral neck recurrence; HPV: human papillomavirus; 3D-CRT: three-dimensional conformal radiation therapy; IMRT: intensity-modulated radiation therapy; brachy: brachytherapy; ECE: extracapsular extension; NR: not reported; CK: CyberKnife.

Study (chronological order)	N	CNR	Nodal details	HPV details	Radiation planning
Jackson et al. (1999) [20]	178	2.2%	Stage	No	3D-CRT (100%)
Kagei et al. (2000) [22]	32	0%	Stage	No	3D-CRT (100%)
O’Sullivan et al. (2001) [7]	228	3.5%	Stage, max dimension	No	3D-CRT (100%)
Jensen et al. (2007) [21]	40	2.5%	Stage	No	3D-CRT (100%)
Rusthoven et al. (2009) [26]	20	0%	Stage	No	3D-CRT (55%), IMRT (45%)
Chronowski et al. (2012) [15]	102	2.0%	Stage, levelⁱ	No	Electron/photon (8.9%), 3D-CRT (25%), IMRT (66%)
Al-Mamgani et al. (2013) [13]	185	1.1%	Stage, nodal levels	No	IMRT + IMRT boost (22%), IMRT + brachy boost (63%), IMRT + CK boost (15%)
Koo et al. (2013) [24]	20	0%	Stage	No	3D-CRT (70%), IMRT (30%)
Lynch et al. (2014) [28]	136	5.9%	Stage, ECE	No	3D-CRT (100%)
Liu et al. (2014) [27]	58	0%	Stage	26% available	3D-CRT (100%)
Hwang et al. (abstract 2014) [19]	46	0%	Stage	No	IMRT (100%)
Cramer et al. (abstract 2014) [16]	23	0%	Stage	No	3D-CRT (96%), IMRT (100%)
Ye et al. (2015) [25]	70	7.1%	Stage	100% available	3D-CRT (NR), IMRT (NR)
Dan et al. (2015) [17]	61	1.6%	Stage	50% available	3D-CRT (23%), IMRT (77%)
Kennedy et al. (2016) [23]	76	1.3%	Stage	12% available	3D-CRT (80%), IMRT (20%)
Rackley et al. (2017)ⁱⁱ [29]	48	0%	Stage, ECE, sizeⁱⁱⁱ	70% available	3D-CRT (NR), IMRT (NR)
Kim et al. (2017) [30]	84	3.6%	Stage, ECE	10% available	3D-CRT (93%), IMRT (7%)
Hu et al. (2017) [18]	37	0%	Stage	Unclear, 62% +	3D-CRT (14%), IMRT (78%), unknown (8%)
Chin et al. (2017)ⁱⁱ [14]	48	0%	Stage, ECE, # of nodes, sizeⁱⁱⁱ	79% available	IMRT (100%)
Huang et al. (2017) [2]	96	2%	Stage, # of nodal levels, # of nodes	100% available	3D-CRT (49%), IMRT (51%)
Maskell et al. (2019) [3]	53	7.5%	Stage, low neck, # of nodal levels (if recurred)	95.6% available	3D-CRT (57%), bilateral neck IMRT (43%)
Current series	37	0%	As above	100% available	IMRT (100%)

**Table 5:** Review of the literature on ipsilateral radiation for oropharyngeal cancer with N2b status ^i ^Study was not included in the analysis as N2b status was not reported; ^ii ^adjuvant study. N2b: N2b staging as per the American Joint Committee on Cancer 7th edition; CNR: contralateral neck recurrence; NR: not reported; RT: radiotherapy; pt: patients.

Study (chronological order)	N2b	% N2b	N2b % CNR w/ ipsilateral RT	CNR, entire cohort
Jackson et al.^i^ (1999) [20]	7 of 178 N2, a-c not specified	3.9%	0 failures (0%) in N2	4 failures of 178 pt (2.2%)
Kagei et al.^i^ (2000) [22]	4 of 32 N2, a-c not specified	12.5%	0 failures (0%) in N2	0 failures of 32 pt (0%)
O’Sullivan et al. (2001) [7]	5 of 228	2.2%	0 failures (0%)	8 failures of 228 pt (3.5%)
Jensen et al.^i^ (2007) [21]	14 of 40 N2, a-c not specified	35.0%	NR	1 failure of 40 pt (2.5%)
Rusthoven et al. (2009) [26]	13 of 20	65%	0 failures (0%)	0 failures of 20 pt (0%)
Chronowski et al. (2012) [15]	22 of 102	21.6%	0 failures (0%)	2 failures of 102 pt (2.0%)
Al-Mamgani et al. (2013) [13]	32 of 185	17.3%	2 failures (6.25%)	2 failures of 185 pt (1.1%)
Koo et al. (2013) [24]	8 of 20	40.0%	0 failures (0%)	0 failures of 20 pt (0%)
Lynch et al. (2014) [28]	55 of 136	40.4%	6 failures (9.2%)	8 failures of 136 pt (5.9%)
Liu et al. (2014) [27]	4 of 58	6.9%	0 failures (0%)	0 failures of 58 pt (0%)
Hwang et al.^i^ (abstract 2014) [19]	35 of 46 N2, a-c not specified	76.1%	0 failures (0%) in N2	0 failures of 46 pt (0%)
Cramer et al. (abstract 2014) [16]	18 of 23	78.3%	0 failures (0%)	0 failures of 23 pt (0%)
Ye et al. (2015) [25]	11 of 70	15.7%	0 failures (0%)	5 failures of 70 pt (7.1%)
Dan et al. (2015) [17]	31 of 61	50.9%	1 failure (3.2%)	1 failure of 61 pt (1.6%)
Kennedy et al. (2016) [23]	26 of 76	34.2%	1 failure (3.8%)	1 failure of 76 pt (1.3%)
Rackley et al. (2017)ⁱⁱ [29]	48 of 256	18.75%	0 failures (0%)	0 failures of 256 pt (0%)
Kim et al. (2017) [30]	38 of 84	45.2%	3 failures (7.9%)	3 failures of 84 pt (3.6%)
Hu et al. (2017) [18]	21 of 37	56.8%	0 failures (0%)	0 failures of 37 pt (0%)
Chin et al. (2017)ⁱⁱ [14]	28 of 48	58.3%	0 failures (0%)	0 failures of 48 pt (0%)
Huang et al. (2017) [2]	8 of 96	8.3%	0 failures (0%)	2 failures of 96 pt (2%)
Maskell et al. (2019) [3]	28 of 53	52.8%	4 failures (14.3%)	4 failures of 53 pt (7.5%)
Current series	26 of 37	70.3%	0 failures pt (0%)	0 failures of 37 pt (0%)
Total	422 of 1590	36.13%	17 of 422 pt (4.03%)	36 failures of 1590 pt (2.3%)

Our cohort includes a relatively high subset of patients that would not widely be considered for unilateral radiation due to multi-nodal (70%) or multi-level nodal involvement (38%). The available literature on multi-level and multi-nodal disease is limited. Of the 21 studies published in the literature on unilateral radiation for oropharyngeal cancer, 17 notated N2b status, two quantified the number of nodes, one described nodal level involvement, and two described the number of nodal levels involved. No other study except the current series reported nodal level involvement, number of nodal levels involved, and number of nodes. Future reports on the treatment of AJCC N2b disease should include further quantification of lymph node burden for further comparison.

With respect to nodal level involvement, Maskell et al. reported that three of the four patients with contralateral neck recurrence had low neck involvement (levels III-IV) but did not report the nodal level involvement for patients without contralateral neck failure [3]. Chronowski et al. considered level IV involvement an institutional contraindication to unilateral neck irradiation; thus, no patients in their series had level IV involvement [15]. In our series, three patients (8%) were found to have level IV/V involvement and 11 patients (30%) with level III/IV/V involvement with no contralateral neck failures. A majority of the involved nodes were in level II (97%), which is consistent with those reported by Al-Mamgani et al.: level II, III, I, IV, and V in 79%, 18%, 14%, 7%, and 2% [13]. Thus, our series likely represents at least a comparable or higher rate of multi-level nodal involvement and low-lying neck disease.

The prognostic utility of the number of nodes and size has previously been described in the post-surgical setting. In patients with resected tonsil cancer, Chin et al. reported that 48% had two to five nodes involved, while 10% of the patients had greater than five nodes involved. No association was identified between recurrence risk and the number of nodes [14]. In our series, 10 (27%) patients had three or more nodes involved at diagnosis. However, quantitative lymph node status was performed radiographically in our series. It is likely that the radiographic number of nodes represents a higher burden of disease as compared to the dissected cohort of Chin et al., as pathologic upstaging is common [31]. Both Chin et al. and Rackley et al. did not identify an association between size (>3 cm) and risk of contralateral recurrence in the postoperative setting [14,29]. Despite these findings, a recent international practice pattern survey revealed that radiation oncologists were more likely to recommend bilateral neck radiation in the setting of multiple involved lymph nodes as compared to surgical oncologists (55% vs. 23%, p < 0.001) [32], which is likely due to the limited available literature. To that end, our study is the first to further quantify radiographic N2b status by both maximal tumor diameter (3.6 cm, IQR: 2.9-4.1 cm) and volumetrically (16 cc, IQR: 10.9-21.8) in the radiographic setting. However, as no patients recurred in our cohort, it was not possible to perform further survival analysis to identify potential risk factors for contralateral neck recurrence. Overall, our low rates of contralateral recurrence even in N2b patients are consistent with the existing literature except for a few series that report moderate rates of contralateral neck failure. Further detailed reporting of nodal level, nodal size, and nodal volume may allow clinicians to further risk-stratify treatment and expand de-escalated unilateral neck irradiation to patients with multi-nodal or multi-level disease.

Aside from the retrospective nature of our study, we were principally limited by a small dataset. Univariate and multivariate analyses were not performed given a lack of recurrences in our cohort. However, our study is one of the few IMRT-exclusive cohorts and the only study to describe nodal level, number, and volume. Our cohort had very limited numbers of patients with (1) lymph node > 6 cm, (2) extranodal extension, and (3) retropharyngeal nodal involvement, which are cases in which ipsilateral radiation is controversial [8]. Thus, our results cannot support ipsilateral neck radiation for these select cases. As our cohort was primarily composed of HPV+ disease, extrapolation to HPV- should be used with caution, although evidence suggests no difference in patterns of failure [2]. Given institutional variations, it is possible that patients in our cohort may be considered to have a lower burden of N2b disease or have had more aggressive upfront staging, which may have led to stage migration. Similarly, the large use of IC (67%), concomitant TFHX (83%), and the use of a response-adapted volume de-escalation (RAVD) paradigm on these contemporary trial parameters [5,10,11,33,34] may limit the generalizability of these findings. However, an induction chemotherapy-based de-escalation approach may be of increasing interest in patients with high-burden diseases, given the concern for high failure rates in this setting in de-escalation setting, as recently published by Ma et al. [35]. Despite these limitations, this is the first study to report nodal volume, level, and number to further characterize the nodal burden of patients with N2b disease. Future directions include continued prospective study of treatment volume de-escalation as well as further study of novel imaging techniques such as single photon emission computed tomography (SPECT)/CT to guide targeted elective nodal irradiation [36,37].

## Conclusions

Overall, these results suggest that patients with well-lateralized tonsil carcinoma and ipsilateral nodal disease may be eligible for ipsilateral radiation regardless of nodal burden. This is the first report demonstrating that nodal volume, level, and number do not seem to have a significant impact on these outcomes. Future studies should provide further detailed information on nodal information beyond the stage to further define the upper limits of de-escalation.

## References

[1] Chaturvedi AK, Anderson WF, Lortet-Tieulent J (2013). Worldwide trends in incidence rates for oral cavity and oropharyngeal cancers. J Clin Oncol.

[2] Huang SH, Waldron J, Bratman SV (2017). Re-evaluation of ipsilateral radiation for T1-T2N0-N2b tonsil carcinoma at the Princess Margaret Hospital in the human papillomavirus era, 25 years later. Int J Radiat Oncol Biol Phys.

[3] Maskell D, Buckley H, Sission K, Roques T, Geropantas K (2019). Ipsilateral neck radiotherapy in N2b well-lateralized tonsil cancer - approach with caution. Head Neck.

[4] Spencer CR, Gay HA, Haughey BH (2014). Eliminating radiotherapy to the contralateral retropharyngeal and high level II lymph nodes in head and neck squamous cell carcinoma is safe and improves quality of life. Cancer.

[5] Seiwert TY, Foster CC, Blair EA (2019). OPTIMA: a phase II dose and volume de-escalation trial for human papillomavirus-positive oropharyngeal cancer. Ann Oncol.

[6] McDowell L, Casswell G, Bressel M (2020). Patient-reported quality of life and toxicity in unilateral and bilateral radiotherapy for early-stage human papillomavirus associated tonsillar carcinoma. Clin Transl Radiat Oncol.

[7] O'Sullivan B, Warde P, Grice B (2001). The benefits and pitfalls of ipsilateral radiotherapy in carcinoma of the tonsillar region. Int J Radiat Oncol Biol Phys.

[8] Tsai CJ, Galloway TJ, Margalit DN (2021). Ipsilateral radiation for squamous cell carcinoma of the tonsil: American Radium Society appropriate use criteria executive summary. Head Neck.

[9] Amdur RJ, Harari PM, Dziegielewski PT, Mendenhall WM (2021). Refining guidelines regarding unilateral treatment in patients with well-lateralized squamous cell carcinoma of the palatine tonsil and multiple positive nodes or extranodal extension. Pract Radiat Oncol.

[10] Villaflor VM, Melotek JM, Karrison TG (2016). Response-adapted volume de-escalation (RAVD) in locally advanced head and neck cancer. Ann Oncol.

[11] Rosenberg AJ, Agrawal N, Pearson A (2021). Risk and response adapted de-intensified treatment for HPV-associated oropharyngeal cancer: Optima paradigm expanded experience. Oral Oncol.

[12] Yom SS, Torres-Saavedra P, Caudell JJ (2021). Reduced-dose radiation therapy for HPV-associated oropharyngeal carcinoma (NRG Oncology HN002). J Clin Oncol.

[13] Al-Mamgani A, van Rooij P, Fransen D, Levendag P (2013). Unilateral neck irradiation for well-lateralized oropharyngeal cancer. Radiother Oncol.

[14] Chin RI, Rao YJ, Hwang MY (2017). Comparison of unilateral versus bilateral intensity-modulated radiotherapy for surgically treated squamous cell carcinoma of the palatine tonsil. Cancer.

[15] Chronowski GM, Garden AS, Morrison WH (2012). Unilateral radiotherapy for the treatment of tonsil cancer. Int J Radiat Oncol Biol Phys.

[16] Cramer CK, Palta M, Patel P, Brizel DM (2014). Ipsilateral tonsil chemoradiation: improved toxicity compared to bilateral radiation and effective rates of local-regional control: definitive management of head-and-neck squamous cell carcinoma. Int J Radiat Biol.

[17] Dan TD, Raben D, Schneider CJ (2015). Freedom from local and regional failure of contralateral neck with ipsilateral neck radiotherapy for node-positive tonsil cancer: updated results of an institutional clinical management approach. Oral Oncol.

[18] Hu KS, Mourad WF, Gamez M (2017). Low rates of contralateral neck failure in unilaterally treated oropharyngeal squamous cell carcinoma with prospectively defined criteria of lateralization. Head Neck.

[19] Hwang MY, Spencer CR, Patel P (2013). Unilateral radiation therapy in node-positive patients with lateralized tonsillar carcinoma: definitive management of head-and-neck squamous cell carcinoma. Int J Radiat Biol.

[20] Jackson SM, Hay JH, Flores AD, Weir L, Wong FL, Schwindt C, Baerg B (1999). Cancer of the tonsil: the results of ipsilateral radiation treatment. Radiother Oncol.

[21] Jensen K, Overgaard M, Grau C (2007). Morbidity after ipsilateral radiotherapy for oropharyngeal cancer. Radiother Oncol.

[22] Kagei K, Shirato H, Nishioka T (2000). Ipsilateral irradiation for carcinomas of tonsillar region and soft palate based on computed tomographic simulation. Radiother Oncol.

[23] Kennedy WR, Herman MP, Deraniyagala RL (2016). Ipsilateral radiotherapy for squamous cell carcinoma of the tonsil. Eur Arch Otorhinolaryngol.

[24] Koo TR, Wu HG (2013). Long-term results of ipsilateral radiotherapy for tonsil cancer. Radiat Oncol J.

[25] Ye A, Bradley KL, Kader H, Wu J, Hay JH (2015). Patterns of relapse in squamous cell carcinoma of the tonsil - unilateral vs. bilateral radiation in the HPV-era. Cureus.

[26] Rusthoven KE, Raben D, Schneider C, Witt R, Sammons S, Raben A (2009). Freedom from local and regional failure of contralateral neck with ipsilateral neck radiotherapy for node-positive tonsil cancer: results of a prospective management approach. Int J Radiat Oncol Biol Phys.

[27] Liu C, Dutu G, Peters LJ, Rischin D, Corry J (2014). Tonsillar cancer: the Peter MacCallum experience with unilateral and bilateral irradiation. Head Neck.

[28] Lynch J, Lal P, Schick U, Nutting CM, Newbold K, Harrington K, Bhide S (2014). Multiple cervical lymph node involvement and extra-capsular extension predict for contralateral nodal recurrence after ipsilateral radiotherapy for squamous cell carcinoma of the tonsil. Oral Oncol.

[29] Rackley TP, Namelo WC, Palaniappan N, Cole N, Owens DM, Evans M (2017). Unilateral radiotherapy for surgically resected lateralized squamous cell carcinoma of the tonsil. Head Neck.

[30] Kim Y, Cho KH, Moon SH (2017). Comparison of the clinical outcomes of patients with squamous cell carcinoma of the tonsil receiving postoperative ipsilateral versus bilateral neck radiotherapy: a propensity score matching analysis (KROG 11-07). Cancer Res Treat.

[31] McMullen CP, Garneau J, Weimar E (2019). Occult nodal disease and occult extranodal extension in patients with oropharyngeal squamous cell carcinoma undergoing primary transoral robotic surgery with neck dissection. JAMA Otolaryngol Head Neck Surg.

[32] de Almeida JR, Seungyeon Kim V, O'Sullivan B (2021). Comparing unilateral vs. bilateral neck management in lateralized oropharyngeal cancer between surgical and radiation oncologists: an international practice pattern survey. Oral Oncol.

[33] Rosenberg A, Agrawal N, Pearson AT (2021). Nivolumab, nabpaclitaxel, and carboplatin followed by risk/response adaptive de-escalated locoregional therapy for HPV-associated oropharyngeal cancer: OPTIMA II trial. J Clin Oncol.

[34] Rosenberg AJ, Izumchenko E, Pearson A (2022). Prospective study evaluating dynamic changes of cell-free HPV DNA in locoregional viral-associated oropharyngeal cancer treated with induction chemotherapy and response-adaptive treatment. BMC Cancer.

[35] Ma DM, Price K, Moore EJ (2021). MC1675, a phase III evaluation of de-escalated adjuvant radiation therapy (DART) vs. standard adjuvant treatment for human papillomavirus associated oropharyngeal squamous cell carcinoma. Int J Radiat Oncol Biol Phys.

[36] de Veij Mestdagh PD, Janssen T, Lamers E (2019). SPECT/CT-guided elective nodal irradiation for head and neck cancer: estimation of clinical benefits using NTCP models. Radiother Oncol.

[37] de Veij Mestdagh PD, Walraven I, Vogel WV (2020). SPECT/CT-guided elective nodal irradiation for head and neck cancer is oncologically safe and less toxic: a potentially practice-changing approach. Radiother Oncol.

